# Biliary Microbiota and Bile Acid Composition in Cholelithiasis

**DOI:** 10.1155/2020/1242364

**Published:** 2020-07-01

**Authors:** Vyacheslav A. Petrov, María A. Fernández-Peralbo, Rico Derks, Elena M. Knyazeva, Nikolay V. Merzlikin, Alexey E. Sazonov, Oleg A. Mayboroda, Irina V. Saltykova

**Affiliations:** ^1^Central Research Laboratory, Siberian State Medical University, Tomsk, Russia; ^2^Department of Analytical Chemistry, Annex Marie Curie Building, Campus of Rabanales, University of Córdoba, E-14071 Córdoba, Spain; ^3^Center for Proteomics and Metabolomics, Leiden University Medical Center, Leiden, Netherlands; ^4^School of Core Engineering Education, National Research Tomsk Polytechnic University, Tomsk, Russia; ^5^Surgical Diseases Department of Pediatric Faculty, Siberian State Medical University, Tomsk, Russia; ^6^Department of Chemistry, Tomsk State University, Tomsk, Russia

## Abstract

**Background:**

A functional interplay between BAs and microbial composition in gut is a well-documented phenomenon. In bile, this phenomenon is far less studied, and with this report, we describe the interactions between the BAs and microbiota in this complex biological matrix. *Methodology*. Thirty-seven gallstone disease patients of which twenty-one with *Opisthorchis felineus* infection were enrolled in the study. The bile samples were obtained during laparoscopic cholecystectomy for gallstone disease operative treatment. Common bile acid composition was measured by LC-MS/MS. Gallbladder microbiota were previously analyzed with 16S rRNA gene sequencing on Illumina MiSeq platform. The associations between bile acid composition and microbiota were analyzed.

**Results:**

Bile acid signature and *Opisthorchis felineus* infection status exert influence on beta-diversity of bile microbial community. Direct correlations were found between taurocholic acid, taurochenodeoxycholic acid concentrations, and alpha-diversity of bile microbiota. Taurocholic acid and taurochenodeoxycholic acid both show positive associations with the presence of Chitinophagaceae family, Microbacterium and Lutibacterium genera, and Prevotella intermedia. Also, direct associations were identified for taurocholic acid concentration and the presence of Actinomycetales and Bacteroidales orders, Lautropia genus, Jeotgalicoccus psychrophilus, and Haemophilus parainfluenzae as well as for taurochenodeoxycholic acid and Acetobacteraceae family and Sphingomonas genus. There were no differences in bile acid concentrations between *O. felineus-*infected and noninfected patients. *Conclusions/Significance*. Associations between diversity, taxonomic profile of bile microbiota, and bile acid levels were evidenced in patients with cholelithiasis. Increase of taurochenodeoxycholic acid and taurocholic acid concentration correlates with bile microbiota alpha-diversity and appearance of opportunistic pathogens.

## 1. Introduction

Liver bile ducts and gallbladder have some of the most unexplored biomes in the human body due to the invasiveness of their exploration. For a long time, bile of the healthy organisms was considered sterile [1], but recently, it has been shown that healthy pigs have a native bile duct microbiota [2], and 16S rRNA gene profiling has confirmed the presence of bacterial amplicons belonging to Firmicutes, Bacteroidetes, and Actinobacteria phyla in the human intact gallbladder bile [3, 4]. Several bile-related disorders can modify the microbiota of the biliary tract and the gallbladder. It was shown that the abundance of Bacteroidaceae, Prevotellaceae, Porphyromonadaceae, and Veillonellaceae families was increased in the bile of patients with cholelithiasis [3]. The primary sclerosing cholangitis leads to a reduction of the microbial diversity with alteration of Pasteurellaceae, Staphylococcaceae, and Xanthomonadaceae abundances, and Streptococcus abundance shows a strong positive correlation with the disease severity and the number of previous cholangiography examinations [5]. Liver fluke infection with *O. viverrini* also resulted in an alteration of taxonomic composition and an increase of alpha-diversity in the bile microbiome in animal model [6]. Human-based study of gallbladder microbiota in *O. felineus* infection confirmed the fact of fluke-induced shifts in the bile microbial community structure and the introduction of taxons undetectable in microbiota of noninfected individuals [7].

Interaction of the gut microbiota with bile acid metabolism is well known. It was reported that the pathology-driven shift in the microbiota diversity may lead to the alterations of the bile acid (BA) repertoire [8]. Alternatively, the BAs themselves can affect gut microbiota community directly (antimicrobial and progerminative actions) and indirectly via farnesoid X receptor activation [9]. The interplay between microbiota and BAs in the bile is much less studied. Current knowledge is mainly limited to the important role of the different levels of BA or its glycine and/or taurine conjugates for the biliary diseases [10, 11]. Liang et al. provided evidence for an association between BA levels and abundance of bacteria from *Bilophila* genus in the supraduodenal segment of common bile duct in cholangiolithiasis patients [12].

In our previous work, we characterized the gallbladder microbiota of patients with gallstone disease [7]. Here, to corroborate additional evidence of the bile pathology-driving changes in gallbladder flora, we are aiming to explore the possible links between the most abundant BAs and microbiota on the background of cholelithiasis.

## 2. Materials and Methods

### 2.1. Study Population

The study was approved by the Ethics Committee of the Siberian State Medical University. Thirty-seven participants (11 males and 26 females) with age ranging from 40 to 61 and diagnosed with gallstone disease were enrolled in the study. Twenty-one of the patients were diagnosed with *O. felineus* infection. Clinical characteristics of the patients are listed in Supplementary Table 1. The bile samples from patients were obtained during surgical gallbladder removal (laparoscopic cholecystectomy). During the surgical intervention, 5–10 milliliters of gallbladder bile were aspirated under sterile conditions and immediately delivered to the laboratory. Two milliliters of bile was clarified by centrifugation (10, 000 g, 10 min, 4°C), the pellet was stored at -80°C for bile microbiota analysis, and the supernatant was stored at -80°C for bile acid analysis.

### 2.2. Bile Acid Analysis

LC-MS/MS analysis was applied for the quantification of the following ten of the most common bile acids (BAs) in a complex matrix such as the bile: cholic acid (CA), chenodeoxycholic acid (CDCA), deoxycholic acid (DCA), ursodeoxycholic acid (UDCA), taurocholic acid (TCA), taurochenodeoxycholic acid (TCDCA), taurolithocholic acid (TLCA), glycocholic acid (GCA), glycochenodeoxycholic acid (GCDCA), and glycodeoxycholic acid (GDCA) as described previously [13]. A detailed description of the analytical procedure is presented in the supplementary material. Briefly, 10 BA reference standards were dissolved in methanol to prepare individual stock solutions. 50 *μ*l of each bile sample from all the patients was collected, mixed to obtain a pool. Bile was 2000-fold diluted using deionized water and incubated with 100 mg/ml activated charcoal for 2 h to strip this matrix of endogenous BAs. The calibration curves were prepared in bile striped from endogenous BAs by treatment with activated charcoal.

For bile samples, C_18_ solid-phase extraction (SPE) cartridges were used for sample clean-up. Bile samples were diluted 2000-fold with LC-MS grade water; 100 *μ*l of diluted bile samples was spiked with 10 *μ*l internal standards, vortexed, and loaded onto preconditioned SPE cartridges. Loaded cartridges were washed with 2 ml H_2_O and eluted with 4 ml MeOH. The eluate was evaporated under vacuum and reconstituted in 100 *μ*l of 50% MeOH. The analysis was carried out by LC-MS/MS using a column in reverse phase in a gradient elution with mobile phases consisted of 0.01% acetic acid in water (mobile phase A) and 0.01% acetic acid in methanol (mobile phase B), at a total flow rate of 0.5 ml/min, in negative ionization mode.

### 2.3. Bile Microbiota Analysis

Gallbladder bile sample microbiota for each of the patient were previously analyzed and described with 16S rRNA gene sequencing on Illumina MiSeq machine [7]. Raw 16S rRNA gene reads data are available on European Nucleotide Archive, accession number PRJEB12755, http://www.ebi.ac.uk/ena/data/view/PRJEB12755. Sequencing results were analyzed as described in Saltykova et al. [7]. Briefly, reads analysis was implemented in QIIME 1.9.0 [14]. After demultiplexing, forward and reverse reads were joined via SeqPrep algorithm; then, reads quality was checked in sliding window with default parameters. Bases with Phred quality score less than 19 were truncated and reads to short after truncation were omitted. The operational taxonomic unit (OTU) picking consisted in usage of the open reference picking strategy by the UCLUST method [15]. Chimera-checked 97% similarity Greengenes taxonomy v13.5 [16] was used as the reference base for taxonomic assignment. All OTUs present only in reagent controls were subtracted from experimental samples to eliminate contamination.

Alpha-diversity or microbial community taxonomic richness was calculated in QIIME using Chao1, observed OTUs, Shannon, and Simpson indices at depth of 200 sequences per sample. For further analysis, we included samples with at least 200 sequences based on sequencing depth and rarefaction curve estimation. All OTUs observed in less than 3 samples were excluded, and microbial data was normalized with CSS algorithm [17]. Distances between samples in unweighted UniFrac metrics for estimation of pairwise dissimilarity between communities (beta-diversity) were calculated in QIIME.

### 2.4. Statistical Analysis

Statistical analysis was implemented in R 3.5.1 version [18]. To examine differences in BA concentrations between infected and noninfected patients, Mann-Whitney-Wilcoxon test was used. FDR-corrected Spearman rank correlation (psych package [19]) was used to define a linkage between taxonomic richness and BA levels. Contribution of BA concentrations to gallbladder flora beta-diversity was estimated with permutational multivariate analysis of variance (algorithm adonis of vegan package [20]) with 9999 permutations in the model which includes distance matrix as outcome and transformed metabolite concentrations along with invasion status and gender as predictors. For this analysis, BA concentrations were transformed with nonmetric multidimensional scaling (NMDS) in Euclidean metrics to one vector that represents the value of the first principle coordinate. NMDS was used for dimension reduction of microbial data to produce scatterplot for visualization of beta-diversity. All metabolites linked with microbiota diversity were enrolled in further analysis. Associations between microbial phylotypes and BA levels were defined by linear regression in model with BA concentrations as outcome, OTU presence data as a predictor, and age, gender, and body mass index as covariates. In case of multiple hypotheses testing, *p* values were corrected with FDR method. Visualization was made in ggplot2 [21] and corrplot [22] packages.

## 3. Results

### 3.1. Gallbladder Bile Acid Signature

Table 1 summarizes the results of the LC-MS-based quantification of the ten most abundant bile acids. The optimized and validated method was applied for the analysis of selected bile acids in the bile of 37 participants. Primary BAs and its conjugates have about 78% of abundance in the gallbladder. The most abundant of measured BAs in human gallbladder with the concentrations more than 1000 ng/ml in all groups were GCA (31.9%), GCDCA (23.6%), GDCA (18.7%), and TCDCA (16.9%). Other measured BAs amount to 9% of total concentration. The high abundance of GCA, GCDCA, and TCDCA in the total bile composition of patients with cholelithiasis was consistent with the results reported for patients with benign biliary disease [23].

### 3.2. Bile Acids and Microbial Community Structure

**Figure 1:**
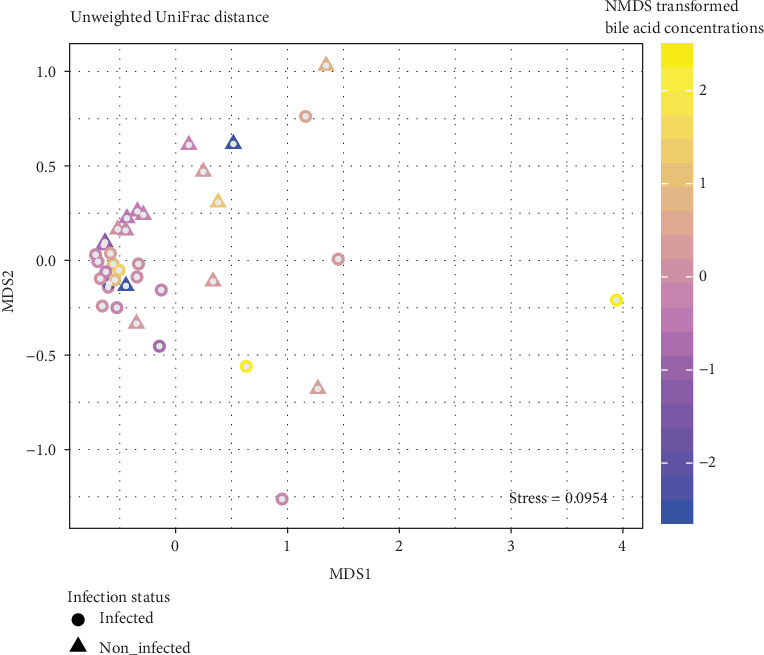
Multidimensional scaling of CSS-corrected OTU abundances in unweighted UniFrac metric. Circle dots represent *O. felineus-*infected samples; triangle dots represent control samples. Color intensity of dots represents the value of the first principal coordinate of MDS-transformed metabolites.

As it was shown recently, *O. felineus* infection affects beta-diversity of the bile flora [7]. Thus, to identify the input of BAs on the microbiota variance, we included in the analysis the status of the infection with the liver fluke. Pairwise dissimilarity between communities (beta-diversity) was computed in unweighted UniFrac metric. BA concentrations were transformed with NMDS to one coordinate vector which was added in adonis model along with *O. felineus* infection status and participants' gender as covariate (Figure 1). It results in 5.8% of microbial data variance explained with *O. felineus* infection status (*p* = 0.006) and 4.6% of variance explained with BAs (*p* = 0.025). Gender does not provide significant input in microbial community structure (*p* = 0.156).

**Figure 2:**
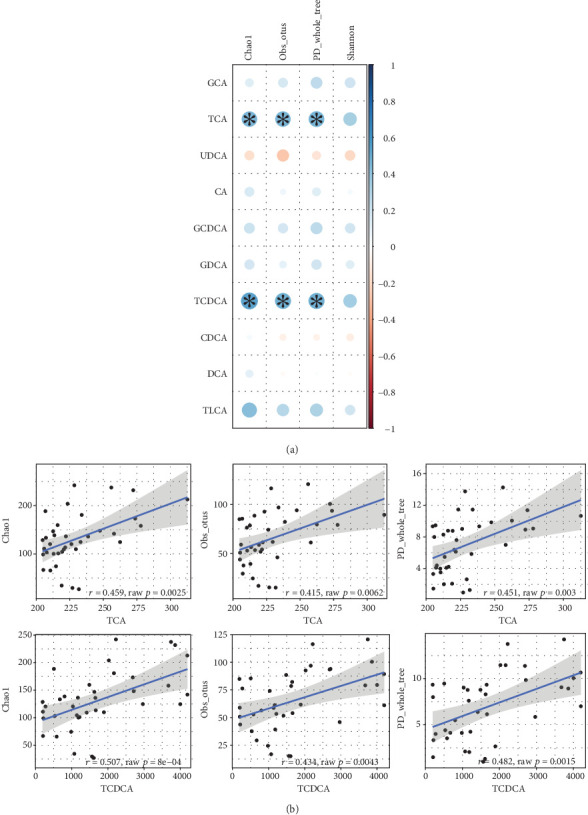
Correlations between alpha-diversity and bile acid concentrations. (a) In the plot, blue circles represent positive correlations, red circles represent negative correlations, the size of circles and its color saturation represent absolute correlation value, and significant correlations (FDR-corrected *p* value < 0.05) are marked with asterisk. (b) In the plot, black line represents regression curve for significant associations, and gray zone represents standard error. CA: cholic acid; CDCA: chenodeoxycholic acid; DCA: deoxycholic acid; UDCA: ursodeoxycholic acid; TCA: taurocholic acid; TCDCA: taurochenodeoxycholic acid; TLCA: taurolithocholic acid; GCA: glycocholic acid; GCDCA: glycochenodeoxycholic acid; GDCA: glycodeoxycholic acid; Obs_otus: observed OTU index.

Taxonomic richness (alpha-diversity) of gallbladder microbiota was estimated at a depth of 200 sequences with richness metrics of Chao1, PD whole tree, Shannon, Simpson, and a number of observed OTUs. To identify possible associations between microbiome alpha-diversity and BA levels, Spearman correlation was used. Significant direct correlations were found between microbial community richness measured with Chao1 and phylogenetically driving PD whole tree indices as well as a number of observed OTUs and the levels of taurine-conjugated forms of primary BAs (TCA and TCDCA, Figure 2).

### 3.3. Associations in the System of Gallbladder Microbiota and Bile Acids

Thus, considering the results presented in Figure 2, TCA and TCDCA were used to test the correlations between BA concentration and appearance of bacterial OTUs in bile. For the analysis, bacterial counts were recomputed to the presence/absence of Boolean values. Linear regression revealed associations of bacterial OTU presence and levels of TCA and TCDCA in gallbladder bile. TCA concentration was directly linked with the presence of Actinomycetales and Bacteroidales orders in gallbladder flora. Chitinophagaceae family, *Lautropia*, *Lutibacterium*, *Microbacterium*, and uncultivated 1-68 genus of (Tissierellaceae) family, and *Jeotgalicoccus psychrophilus*, *Prevotella intermedia*, and *Haemophilus parainfluenzae* species also were linked with TCA concentration (Table 2, S1 Fig). TCDCA concentration shows positive associations with the presence of OTUs belonging to Chitinophagaceae and Acetobacteraceae families, *Microbacterium, Lutibacterium*, and *Sphingomonas* genera, and *Prevotella intermedia* species (Table 2, S2 Fig).

### 3.4. Bile Acid Concentrations and *O. felineus* Infection

**Figure 3:**
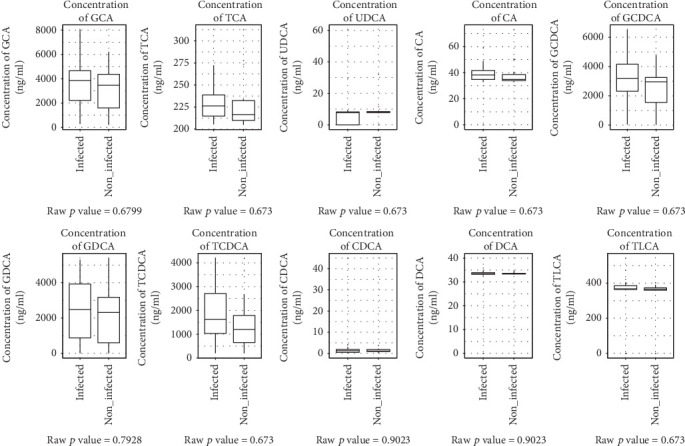
Concentrations of bile acids in gallbladder bile samples. On the plot boxes, patients with *O. felineus* infection (infected) and patients without infection with *O. felineus* (noninfected) are represented. Whiskers' length represents 1.5 of interquartile range. CA: cholic acid; CDCA: chenodeoxycholic acid; DCA: deoxycholic acid; UDCA: ursodeoxycholic acid; TCA: taurocholic acid; TCDCA: taurochenodeoxycholic acid; TLCA: taurolithocholic acid; GCA: glycocholic acid; GCDCA: glycochenodeoxycholic acid; GDCA: glycodeoxycholic acid.

The results of metabolomics analysis of *O. felineus* infection in animal model show that urinary metabolic profiles of the experimental animal change considerably and the urinary BAs are among the main factors explaining the effect [24]. Thus, to investigate the role of the infection status on BA composition in gallbladder disease, we analyzed the measured BAs with respect to the infection status of the patients. There were no significant differences in BA level (Figure 3), total gallbladder BA concentration (*p* = 0.24), and primary to secondary BA ratio (*p* = 0.59) between *O. felineus-*infected patients and control group.

## 4. Discussion

A functional interplay between BAs and microbial composition in gut is a well-documented phenomenon [25]. In the bile, this phenomenon is far less studied, and with this report, we describe the interactions between the BAs and microbiota in this complex biological matrix. Collecting the material for this study, we could not avoid inclusion of the patients with *O. felineus* infection; thus, it is only logical that we stress a possible influence of the infection on BAs and bile microbiota community. The role of *O. felineus* infection in the bile microbiota composition was proposed and discussed by Saltykova et al. [7]. We have shown that *O. felineus* infection has no strong effect on the BA profile in patients with cholelithiasis. Yet, the infection influences the gallbladder microbiota beta-diversity.

Furthermore, we reported significant direct correlations between TCA and TCDCA and the bile microbiota alpha-diversity. TCA concentration was associated with the appearance of species *Jeotgalicoccus psychrophilus*, *Prevotella intermedia*, and *Haemophilus parainfluenzae* in the bile. TCDCA concentration shows positive associations with the presence of OTUs belonging to *Microbacterium*, *Lutibacterium*, and *Sphingomonas* genera and *Prevotella intermedia* species.

In our study, we observed correlations between primary BAs and bile bacteria, while fecal microbiota disturbance was associated mostly with secondary BAs in feces. Specifically, the analysis of BAs and fecal microbiota in gallstone patients revealed that genus *Oscillospira* was positively correlated with the fraction of secondary BAs; this association is attributed to the association of *Oscillospira* and relative fraction of lithocholic acid in the feces [26]. The positive correlation between bacterial taxa and secondary BAs was observed for patients with alcoholic cirrhosis and severe alcoholic hepatitis [27]. It was hypothesized that microbiota and secondary BA correlations were observed due to the role of the gut flora in the direct or indirect conversion of primary BAs to secondary BAs [28]. Here, we observed correlations between primary BAs and bile microbiota that may be related to different mechanisms of the selective force of BAs for bile and gut microbiota.

It is worth of mentioning that TCA and TCDCA associated with bile microbiota diversity and composition are also known as the markers of liver injury and/or liver dysfunction. A recent report of Luo et al. indicates a possible role of TCA as a maker of the liver impairment [28]. Metabolomics studies demonstrated that TCA and TCDCA concentration was elevated in serum of liver cirrhotic patients and positively correlated with Child–Pugh scores [29]. *In vitro* experiments indicated that exposure to TCDCA increases expression of the c-myc oncoprotein in WRL-68 cell (hepatocyte like morphology) and downregulates expression CEBP*α* tumor suppressor protein in HepG2 cells (epithelial morphology) [30]. In a mouse model of hepatocellular carcinoma, augmentation of TCDCA intestinal excretion prevented carcinoma development [30].

Most of the bacteria associated with TCA and TCDCA concentrations are treated as opportunistic pathogens. *Haemophilus parainfluenzae* is a common liver pathogen. It was found to be more abundant in fecal microbiome of biliary cirrhosis patients [31] and was isolated from bile samples of patients with acute cholecystitis [32] and liver abscesses [33]. *Microbacterium* genus abundance was associated with different inflammatory disorders: otitis externa [34], noma [35], and bacteremia [36]. OTUs belonging to (Tissierellaceae) family were more abundant in ulcerative colitis sites [37]. *Prevotella intermedia* was identified in atopic liver abscess and in case of periodontitis [38].

In conclusion, associations between diversity, taxonomic profile of bile microbiota, and bile BA levels were evidenced in patients with cholelithiasis. The increase of TCDCA and TCA concentration correlates with bile microbiota alpha-diversity and appearance of opportunistic pathogens in bile of patients with cholelithiasis.

## Figures and Tables

**Table 1:** Gallbladder bile acid signature of analyzed bile samples.

Bile acid	Concentration, median [Q1; Q3] (ng/ml)
Glycocholic acid (GCA)	3620.52 [2006.03; 4579.32]
Glycochenodeoxycholic acid (GCDCA)	3098.43 [1820.37; 4033.03]
Glycodeoxycholic acid (GDCA)	2406.45 [707.73; 3285.40]
Taurochenodeoxycholic acid (TCDCA)	1486.25 [801.10; 2221.75]
Taurolithocholic acid (TLCA)	366.71 [362.42; 385.20]
Taurocholic acid (TCA)	221.61 [212.60; 233.91]
Cholic acid (CA)	36.67 [33.97; 41.39]
Deoxycholic acid (DCA)	33.47 [33.23; 33.90]
Ursodeoxycholic acid (UDCA)	7.87 [0; 8.37]
Chenodeoxycholic acid (CDCA)	1.27 [0.80; 1.83]

**Table 2:** Associations between BA levels and microbial OTU abundances.

Bile acid	Taxonomy	Beta	Standard error	*p* value	Adj. *p* value
TCA	Actinomycetales	41.90	10.78	0.0006	0.0286
TCA	*Microbacterium*	40.69	11.52	0.0015	0.0396
TCA	Bacteroidales	84.87	22.18	0.0007	0.0286
TCA	*Prevotella intermedia*	28.81	8.37	0.0019	0.0465
TCA	Chitinophagaceae	54.63	9.89	7.51*E* − 006	0.0028
TCA	*Jeotgalicoccus psychrophilus*	47.64	12.96	0.0010	0.0318
TCA	1-68 of (Tissierellaceae)	48.09	12.82	0.0009	0.0313
TCA	*Lutibacterium*	46.67	12.15	0.0007	0.0286
TCA	*Lautropia*	64.86	14.15	9.30*E* − 005	0.0114
TCA	*Haemophilus parainfluenzae*	84.87	22.18	0.0007	0.0286
TCDCA	*Microbacterium*	2114.47	550.69	0.0007	0.0286
TCDCA	*Prevotella intermedia*	1548.85	393.14	0.0005	0.0286
TCDCA	Chitinophagaceae	2608.39	501.50	1.77*E* − 005	0.0033
TCDCA	Acetobacteraceae	2399.28	652.76	0.0010	0.0318
TCDCA	*Lutibacterium*	2178.51	613.07	0.0014	0.0396
TCDCA	*Sphingomonas*	1310.20	386.61	0.0022	0.0499

Beta represents regression line's slope coefficient, SE represents beta's standard error, and adj. *p* value represents FDR-corrected *p* value.

## Data Availability

Raw 16S rRNA gene reads data are available on European Nucleotide Archive, accession number PRJEB12755, http://www.ebi.ac.uk/ena/data/view/PRJEB12755.

## Abbreviations

BAs: Bile acids
LC-MS/MS: Liquid chromatography-mass spectrometry and tandem mass spectrometry
TCA: Taurocholic acid
TCDCA: Taurochenodeoxycholic acid
OTUs: Operational taxonomic units
CA: Cholic acid
CDCA: Chenodeoxycholic acid
DCA: Deoxycholic acid
UDCA: Ursodeoxycholic acid
TLCA: Taurolithocholic acid
GCA: Glycocholic acid
GCDCA: Glycochenodeoxycholic acid
GDCA: Glycodeoxycholic acid
DCA-d4: Deoxycholic acid d-4
NMDS: Nonmetric multidimensional scaling.

## Supplementary Materials

Supplementary Materials Table 1: brief clinical characteristics of the patients enrolled in the study including age, gender, and infection status of *Opisthorchis felineus* and the presence of other hepatobiliary diseases (Table 1 was published in Saltykova IV, Petrov VA, Logacheva MD, et al. Biliary Microbiota, Gallstone Disease and Infection with Opisthorchis felineus. PLoS Negl Trop Dis. 2016; 10(7):e0004809. Published 2016 Jul 22. doi: 10.1371/journal.pntd.0004809). S1 Fig: associations between TCA levels and microbial OTU abundances. S2 Fig: associations between TCDCA levels and microbial OTU abundances.
