# Supplementary material for: Biliary Microbiota and Bile Acid Composition in Cholelithiasis
Source: Biomed Res Int. 2020 Jul 1;2020:1242364. doi: 10.1155/2020/1242364 (PMC7352139; doi:10.1155/2020/1242364)
Supplement: Supplementary Materials — Table 1: brief clinical characteristics of the patients enrolled in the study including age, gender, and infection status of Opisthorchis felineus and the presence of other hepatobiliary diseases (Table 1 was published in Saltykova IV, Petrov VA, Logacheva MD, et al. Biliary Microbiota, Gallstone Disease and Infection with Opisthorchis felineus. PLoS Negl Trop Dis. 2016; 10(7):e0004809. Published 2016 Jul 22. doi: 10.1371/journal.pntd.0004809). S1 Fig: associations between TCA levels and microbial OTU abundances. S2 Fig: associations between TCDCA levels and microbial OTU abundances. [file 1242364.f1.docx]

Supplementary material

Table 1

Brief clinical characteristics of the patients enrolled in the study, including age, gender, and infection status of *Opisthorchis felineus* and presence of other hepatobiliary diseases (Table 1 was published in the Saltykova IV, Petrov VA, Logacheva MD, et al. Biliary Microbiota, Gallstone Disease and Infection with Opisthorchis felineus. PLoS Negl Trop Dis. 2016;10(7):e0004809. Published 2016 Jul 22. doi:10.1371/journal.pntd.0004809)

| Variable | Infected with *Opisthorchis felineus* | Not-infected with *Opisthorchis felineus* |
| --- | --- | --- |
| Cholelithiasis | 21 | 16 |
| Median age (IQR) | 57.5 (42.5-59.5) | 55 (41-60) |
| Male/Female | 6/15 | 5/11 |
| Cholelithiasis & pancreatitis | 1 | 1 |
| Cholelithiasis & hepatitis C virus | 1 | 1 |

Bile acid analysis

The following ten of the most common bile acids (BAs) in a complex matrix such as the bile were analyzed: cholic acid (CA), chenodeoxy cholic acid (CDCA), deoxycholic acid (DCA), ursodeoxycholic acid (UDCA), taurocholic acid (TCA), taurochenodeoxycholic acid (TCDCA), taurolithocholic acid (TLCA), glycocholic acid (GCA), glycochenodeoxycholic acid (GCDCA), glycodeoxycholic acid (GDCA) and deoxycholic acid d-4 (DCA-d4) as internal standard.

The analysis was carried out by LC-MS/MS using a column in reverse phase in a gradient elution with mobile phases consisted of 0.01% acetic acid in water (mobile phase A) and 0.01% acetic acid in methanol (mobile phase B), at a total flow rate of 0.5 ml/min, in negative ionization mode.

The 10 BAs reference standards were dissolved in methanol to prepare individual stock solutions. Bile was 2000-fold diluted using deionized water and incubated with 100mg/ml activated charcoal for 2h to strip this matrix of endogenous BAs. Mixture was centrifuged at 13,000×g for 10min twice, and the supernatant was filtered. The calibration curves were prepared in bile striped from endogenous BAs by treatment with activated charcoal.

For bile samples, C_18_ Solid-phase extraction (SPE) cartridges were used for bile acids extraction. Bile samples were diluted 2000-fold with LC-MS grade water, a 100µl of diluted bile samples was spiked with 10µl internal standart, vortexed, and loaded onto SPE cartridges pre-conditioned with 2ml MeOH, followed by 2ml H2O. Loaded cartridges were washed with 2ml H2O and eluted with 4ml MeOH. The eluate was evaporated under vacuum and reconstituted in 100µl of 50%MeOH.

15 µl of each bile samples from all the patients were collected, mixed to obtain a pool, aliquoted and stored as quality control (QC) samples to be daily analyzed to ascertain that the mass spectrometer performance was stable during the analysis of the samples set. Thus, 3 QCs were run per day following the same protocol as that used for samples.

The methods was properly assessed by evaluation of their analytical features and then applied to real samples. As matrix constituents influence the extraction process, the recovery study was performed using untreated bile instead of a charcoal treated bile in order to have extraction conditions as close as possible to those of real samples. In order to assess efficiency of the extraction procedure, six bile QC samples were extracted for each a low, medium and a high concentration (10, 300 and 1000 ng/ml). Recovery was obtained by expressing the mean peak area of samples spiked before extraction as a percentage of that of samples spiked after extraction. The extraction recoveries for bile acids ranged from 58.7% to 96.2% and were slightly higher for 1000 ng/ml for almost all of the compounds.

To assess matrix effects, three QC were extracted and spiked with low, medium, and high amounts of standard stock solutions (50, 500, and 1000 ng/ml for BAs). Simultaneously, identical amounts of standard stock solutions and internal standard were pipetted into three clean vials. Quantitative matrix effects were assessed by expressing the peak area of spiked samples on the peak area of samples contained in standard solutions. The signal in presence of matrix components was between 100.0% and 116.1% of the signal in pure standards for all analytes, which indicated some competition between the analytes and interfering matrix components.

The precision of the method was estimating intra and inter-day variability and calculated as relative standard deviation (RSD) and expressed as percentage. For bile acids, intra-day (inter-day) variation (n=3) ranged from 0.13% to 9.82% (2.04% to 12.33%).

**S1 Fig. Associations between TCA levels and microbial OTUs abundances**

**S2 Fig. Associations between TCDCA levels and microbial OTUs abundances**
